# Editorial: Decoding immune heterogeneity: therapeutic responses and resistance in diverse cellular landscapes

**DOI:** 10.3389/fphar.2026.1908327

**Published:** 2026-07-08

**Authors:** Jianzhi Wang, Peiliang Xie, Manting Wang, Dawei Chen

**Affiliations:** 1 Department of Orthopedic Surgery and Orthopedic Research Institute, West China Hospital, Sichuan University, Chengdu, China; 2 West China School of Medicine, Sichuan University, Chengdu, China; 3 Institute of Clinical Molecular Biology, University Hospital Schleswig-Holstein, Kiel University, Kiel, Germany

**Keywords:** immune heterogeneity, immunopharmacology, inflammation, precision medicine, single-cell analysis, therapeutic responses, tumor microenvironment

## Introduction

Why can the same intervention help one patient, fail in another, or cause harm? Single-cell and multi-omics studies have made this old pharmacological question more precise (Stuart and Satija, 2019). Cells assigned to the same immune lineage may differ substantially in function because of their tissue location, differentiation history, metabolic state, treatment exposure, or disease stage (Lahnemann et al., 2020). Methods that resolve and integrate these states have made molecularly informed patient selection more plausible. They have also shown how much biological Research Topic can sit behind a single clinical diagnosis (Argelaguet et al., 2021; Hamburg and Collins, 2010).

The Research Topic “Decoding Immune Heterogeneity: Therapeutic Responses and Resistance in Diverse Cellular Landscapes” follows this problem across inflammatory disease, cancer, neuroimmune injury, biomaterial-associated immune responses, and drug safety. Its contributions range from mechanistic experiments and single-cell analyses to prognostic modeling and pharmacovigilance. The methods are diverse because the underlying question appears at several scales: cellular state, local tissue organization, patient Research Topic, and treatment exposure.

Our aim here is to read these papers together. The clearest links arise where a biological difference is connected to an intervention and an observable result, such as treatment benefit, resistance, tissue repair, or an adverse-event signal. The limits are just as informative. A molecular association may suggest a mechanism without establishing one, and a predictive model may perform well in its development dataset without being ready for clinical use.

## Immune heterogeneity in inflammatory and immune-mediated diseases

Inflammatory diagnoses remain clinically necessary, but they can group together patients whose disease is sustained by different immune processes (Liu et al., 2025). Across this part of the Research Topic, T cell differentiation, local metabolism, non-coding RNA regulation, and neuroimmune signaling are considered as possible sources of that Research Topic. The articles are therefore linked less by organ system than by the question of which process is actually driving inflammation in a given setting.

At the autoimmune end of the Research Topic, Zhu et al. examine T cell subset imbalance in primary biliary cholangitis and the therapeutic opportunities that may follow from altered differentiation and function. Zhao et al. approach immune regulation through metabolism and delivery: in an experimental periodontitis model, a thermosensitive hydrogel carrying 2-deoxy-D-glucose suppresses glycolysis and the Th17 effector response. Read together, these studies suggest that choosing a pathway and choosing where to intervene in that pathway may be inseparable.

The same problem appears in different form in acute pancreatitis and osteoarthritis. Chen et al. assess the long non-coding RNA AC245100.4 and discuss its potential diagnostic, prognostic, and inflammation-related relevance. Liu et al. consider how immune-state differences may influence responses to platelet-rich plasma and TRPV1 antagonism in osteoarthritis, bringing immune and neuroimmune mechanisms into a disorder often described mainly in structural terms. These articles do not yet supply a shared test for patient selection. They do identify the next practical question: can the relevant T cell, metabolic, RNA, or neural-immune feature be measured before treatment and linked reproducibly to response?

## Immune ecosystems, therapeutic resistance, and computational modeling in cancer

Cancer makes the multicellular nature of treatment failure difficult to ignore. Resistance may arise within the malignant cell or through signals supplied by other cells (Sharma et al., 2017). Stromal populations, immune cells, cytokines, and tissue organization can all alter the response to therapy (Binnewies et al., 2018; Meng et al., 2026). In immunotherapy, this means that the tumor and its immune microenvironment often need to be considered as one functional system (Xiong et al., 2024; Meng et al., 2024).

Ma et al. address that interaction directly, reporting that mesenchymal stem cells promote cisplatin resistance in non-small cell lung cancer through an IL-6/MEK-ERK/macrophage axis. Their experimental work places resistance in the exchange among several cell populations. A computational route is taken by the two studies centered on immunogenic cell death. Xu et al. characterize associated Research Topic in osteosarcoma and test a prognostic model experimentally. Wang et al. develop a multi-omics, machine learning-based model in colon cancer and examine the functional relevance of FCGR2A.

These models should be judged by a sequence of questions rather than by a general call for more validation. Are the selected variables interpretable? Does the model retain useful performance in a genuinely independent population? Does it add information beyond routine clinical factors? Does it nominate biology that can be tested experimentally? The papers in this group begin to address the first and last questions. The middle two will determine whether the models remain research tools or eventually contribute to treatment decisions.

## Context-dependent immune vulnerability, biomaterials, and pharmacovigilance

The remaining articles appear disparate at first: neonatal neurotoxicity, neurodegeneration, nutrition in preterm infants, a bone-repair material, and post-marketing drug safety. Their connection lies in the conditions surrounding an immune response. Developmental stage, redox balance, nutritional exposure, a material interface, or the pattern of drug use can change what a biological signal means and how confidently it can be interpreted.

Huang et al. study ferroptosis-related vulnerability in isoflurane-induced neonatal neurotoxicity through the SLC7A11/GPX4 axis. Knudsen et al. consider redox regulation over a longer disease course, reviewing glutathione in neurodegenerative disease and its relation to oxidative stress, immune dysfunction, and neuronal injury. Developmental and nutritional conditions are also central to the secondary analysis by Zhuang et al., which examines a partially hydrolyzed formula with high sn-2 palmitic acid in preterm infants. In each case, the immune measure is interpreted against a specific stage of development or exposure history rather than as an isolated marker.

Biomaterial-associated repair provides another local context for interpreting immune signals. At the material–tissue interface, inflammatory cues, cellular communication, and repair pathways need to be considered together. In this sense, the material can be viewed as part of an active healing microenvironment rather than as a passive scaffold. Lin et al. move to the population level by analyzing bimekizumab reports in the FDA Adverse Event Reporting System. Such databases are useful for detecting reporting signals, but they cannot estimate incidence or prove causality on their own (Edwards and Aronson, 2000). Read alongside the studies above, this pharmacovigilance analysis illustrates the breadth of settings in which immune signals must be interpreted in relation to their surroundings, including communication among cells in local tissues (Armingol et al., 2021).

## Conclusion

Taken as a Research Topic, these papers use immune Research Topic to explain differences in therapeutic effect rather than merely to describe cell populations. Metabolic modulation in periodontitis, stromal support of cisplatin resistance, models of immunogenic cell death, immune responses to a biomaterial, and post-marketing surveillance all locate treatment response at a different biological scale. No single biomarker or analytical method emerges as a general solution, nor should one be expected from such different disease settings.

The boundaries of the evidence are equally important. Cell-state findings must survive changes in cohort and analytical pipeline. Computational predictions need experimental support, and safety signals require confirmation with data that can address incidence and causality. Molecular profiling may improve patient selection in some settings, but the added measurement is worthwhile only when it can change a decision. Individualized immune profiling is therefore likely to be selective rather than routine. The papers in this Research Topic help clarify where that selectivity may matter.
